# Beyond *Candida*: Epidemiological Insights into Rare Yeast Fungaemia in Greece from a 15-Year Hospital-Based Study and Literature Review

**DOI:** 10.3390/jof12030187

**Published:** 2026-03-05

**Authors:** Maria Siopi, Dimitrios Mitsopoulos, Angeliki Alevra, Margarita Vargiami, Spyros Pournaras, Joseph Meletiadis

**Affiliations:** 1 Clinical Microbiology Laboratory, “Attikon” University General Hospital, Medical School, National and Kapodistrian University of Athens, Rimini 1, 12462 Athens, Greece; marizasiopi@hotmail.com (M.S.); mvargiami@hotmail.com (M.V.);; 2 Molecular Microbiology and Immunology Laboratory, Department of Biomedical Sciences, University of West Attica, Agiou Spiridonos 28, 12243 Athens, Greece; dimitris.mits01@gmail.com (D.M.); angelikealeura@gmail.com (A.A.)

**Keywords:** fungaemia, rare yeasts, epidemiology, Greece

## Abstract

Invasive infections attributed to rare yeasts are increasingly recognized and often exhibit resistance to echinocandins or fluconazole. Despite geographical variability, epidemiological data from Greece are limited. To bridge this gap, we conducted a 15-year retrospective study of rare yeast fungaemia at a tertiary teaching hospital in Athens. All microbiologically confirmed cases in hospitalized patients (2010–2024) were included. Demographic and clinical data were retrieved from medical records. Incidence was calculated per 1000 admissions and 10,000 bed days. Isolates were identified using Vitek 2 and, when available, further characterized by MALDI-TOF MS and tested for antifungal susceptibility based on EUCAST guidelines. A total of 29 episodes (3% of all fungaemias) were identified, with an incidence of 0.04/1000 admissions and 0.09/10,000 bed days. Hematological malignancies (31%) were the most common underlying condition. All patients had central venous catheters and were receiving antibiotics; 38% were non-neutropenic/non-immunosuppressed. The most frequently isolated species were *Rhodotorula mucilaginosa* (41%), *Saccharomyces cerevisiae* (31%) and *Trichosporon asahii* (21%), with single cases of *Apiotrichum loubieri* and *Magnusiomyces capitatus.* Amphotericin B exhibited good in vitro activity against all species; echinocandins were active only against *S. cerevisiae*, and azole activity was species dependent. The median time from hospital admission to fungaemia onset was 27 days; one-third of cases were breakthrough infections, and in 19%, diagnosis was established post-mortem. Despite antifungal therapy, crude mortality was 53%. Although infrequent, rare yeast fungaemia at our centre was associated with high breakthrough rates and substantial mortality. Regional epidemiological insight and heightened clinical awareness are key to optimizing outcomes.

## 1. Introduction

In recent years, invasive fungal infections caused by rare pathogens have drawn growing clinical attention, largely due to their association with high morbidity and mortality in immunocompromised populations [1]. Rare yeast fungaemias are typically defined as bloodstream infections (BSIs) caused by yeast species other than *Candida* and *Cryptococcus* genera [2]. Of note, due to recent taxonomic revisions, several species historically classified as *Candida* spp. have been reassigned to other genera [3]. For clarity, we therefore excluded both species currently classified as *Candida* spp. and those formerly assigned to *Candida* genus but now placed in other genera. Their epidemiology is multifactorial, shaped by diverse host-related and healthcare-associated factors, and exhibits considerable regional variation that likely reflects differences in antifungal prophylaxis practices, diagnostic capabilities and patient demographics [2,4].

Rare yeast fungaemias pose significant diagnostic and therapeutic challenges. Laboratory diagnosis remains difficult, as many rare yeasts exhibit atypical phenotypic features and may be misidentified using conventional methods. Limited access to advanced techniques, such as matrix-assisted laser desorption/ionization-time of flight mass spectrometry (MALDI-TOF MS) and molecular identification, as well as the lack of surrogate biomarkers further impede timely and accurate identification [4,5]. As such, and given that the diagnosis of rare yeast fungaemia relies on a high index of suspicion, infections caused by these rare pathogens may be misdiagnosed or diagnosed late, hindering appropriate and timely therapeutic intervention [6]. In addition, therapeutic management is complicated by the intrinsic resistance or reduced susceptibility of many rare yeasts to echinocandins and fluconazole, contributing to suboptimal outcomes and an increased risk of breakthrough infections [4]. Meanwhile, interpretation of antifungal susceptibility testing (AFST) results is constrained by the absence of established clinical breakpoints for these pathogens [5,7].

In light of these drawbacks, epidemiological investigation of rare yeast fungaemia is essential, especially amid widespread empirical antifungal use and evolving fungal ecology. Nonetheless, existing data are largely restricted to case reports, small case series and single-centre studies, often focusing on specific pathogens or patient populations, thereby limiting the generalizability of findings. Moreover, the global distribution and incidence of the infection is unclear, as efforts to extrapolate geographical prevalence are hindered by factors such as variable diagnostic capacity and awareness [2,4]. This scarcity of comprehensive epidemiological data has impeded a thorough understanding of rare yeast fungaemia and highlights the need for broader, systematic surveillance. At the national level, the epidemiological landscape in Greece remains largely undefined.

Herein, we aim to address this knowledge gap by reviewing all nationally reported cases from Greece, along with a comprehensive overview of the burden, microbiological profiles, and clinical characteristics of rare yeast fungaemias diagnosed over a 15-year period at a tertiary academic hospital in Athens, thereby informing clinical decision making and guiding future surveillance efforts.

## 2. Materials and Methods

**Literature review.** An electronic search was performed in October 2025 across PubMed, Google Scholar, and Web of Science databases, using the keywords ‘*Apiotrichum*’, ‘*Blastoschizomyces*’, ‘bloodstream’, ‘*Cutaneotrichosporon*’, ‘*Cystobasidium*’, ‘*Dipodascus*’, ‘*Dirkmeia*’, ‘disseminated’, ‘fungaemia’, ‘*Galactomyces*’, ‘geotrichosis’, ‘*Geotrichum*’, ‘*Kodamaea*’, ‘*Lodderomyces*’, ‘*Magnusiomyces*’, ‘*Malassezia*’, ‘*Moesziomyces*’, ‘*Pityrosporum*’, ‘*Pseudozyma*’, ‘*Rhodotorula*’, ‘*Saccharomyces*’, ‘*Saprochaete*’, ‘*Sporobolomyces*’, ‘*Sporopachydermia*’, ‘*Trichosporon*’, and ‘trichosporonosis’ combined with ‘Greece’ and/or ‘Greek’. The reference lists of the retrieved articles were manually reviewed to identify additional relevant studies. Only English-language articles were included without restrictions regarding publication date. Data extracted from eligible studies included (when available): study setting and period, patient demographics (age and sex) and clinical background (underlying conditions and risk factors), causative agents and their antifungal susceptibility profiles, antifungal treatment regimens, and outcome.

**Study setting.** A retrospective surveillance study was conducted at the Attikon University General Hospital to identify cases of rare yeast fungaemia over a 15-year period, from 2010 to 2024. Attikon is a modern 750-bed tertiary academic hospital located in the region of Attica, which includes the greater Athens metropolitan area and represents the most densely populated area in Greece. The hospital comprises a wide range of specialized departments, including adult, pediatric, and neonatal intensive care units (ICUs), hematology and oncology wards, as well as dedicated units for bone marrow transplantation and the management of HIV/AIDS patients.

Rare yeast fungaemia was defined as the isolation of a yeast species, excluding *Candida* spp. and *Cryptococcus* spp. [2], from at least one blood culture obtained during hospitalization, accompanied by clinical signs and symptoms of systemic infection, such as fever and/or systemic inflammatory response syndrome or sepsis. Demographic characteristics (age and sex), the hospital unit at the time of infection onset, underlying conditions, mycological findings and patient outcomes were retrieved from the hospital’s computerized databases. Information regarding antifungal treatment was collected through manual review of paper-based medical records (when available). Prolonged neutropenia was defined as an absolute neutrophil count of <500 cells/μL persisting for at least 10 consecutive days. Prior antifungal exposure was considered present if the patient had received antifungal therapy for ≥7 days when there was first clinical suspicion of fungaemia. Prolonged ICU stay was defined as an ICU stay of ≥14 days within the 30 days preceding the diagnosis of infection. In the surgical history, major surgical procedures performed within the month prior to the onset of fungaemia were evaluated.

**Identification.** Initial species identification of recovered isolates was performed using biochemical phenotyping with the Vitek 2 automated system according to the manufacturer’s instructions (Vitek 2-YST; bioMérieux, Marcy l’Etoile, France). For stored strains (preserved in sterile saline with 10% glycerol at −70 °C), species-level confirmation was retrospectively conducted by MALDI-TOF MS (Bruker Daltonics, Bremen, Germany). In cases where MALDI-TOF MS yielded inconclusive results or the organism was not included in the reference library (IVD and BDAL revision 11), sequencing of the internal transcribed spacer (ITS) region was performed using ITS1 (5-TCCGTAGGTGAACCTGCGG-3) and ITS4 (5-TCCTCCGCTTATTGATATGC-3) primers, as previously described [8].

**AFST**. In vitro AFST was carried out following the European committee for antimicrobial susceptibility testing (EUCAST) broth microdilution reference methodology (E.Def 7.4) [9]. The antifungal agents tested included amphotericin B, fluconazole, posaconazole, itraconazole, isavuconazole (all from Sigma-Aldrich, Athens, Greece), voriconazole (Pfizer Ltd., Kent, UK), anidulafungin (Pfizer, Groton, CT, USA), micafungin (Astellas Pharma, Tokyo, Japan) and caspofungin (Merck and Co., Whitehouse, NJ, USA). Final drug concentrations ranged from 0.06 to 8 mg/L for amphotericin B, from 0.5 to 64 mg/L for fluconazole, and from 0.008 to 8 mg/L for the remaining antifungals. The recommended quality control strains *Pichia kudriavzevii* (formerly *C. krusei*) ATCC 6258 and *C. parapsilosis* ATCC 22019 were included. Microtitration plates were incubated at 35 ± 2 °C for 24 h and minimum inhibitory concentrations (MICs) were assessed spectrophotometrically (540 nm). The MIC for amphotericin B was defined as the lowest drug concentration resulting in ≥90% inhibition of fungal growth compared to the drug-free control, whereas for all other antifungals, the MIC corresponded to the lowest drug concentration leading to ≥50% inhibition compared to the growth control. Due to the absence of clinical breakpoints, antifungal susceptibility profiles were interpreted in accordance with the EUCAST epidemiological cut-off values (ECOFFs), when available [10], and following EUCAST guidance for MIC interpretation for rare yeasts without species-specific breakpoints, limited to species and antifungal agents included in current recommendations [11].

**Analysis.** The annual incidence of rare yeast BSIs was determined by dividing the number of rare yeast fungaemia episodes by the total number of fungaemia episodes recorded each year. Incidence rates were additionally expressed per 1000 hospital admissions and per 10,000 patient bed days, using data extracted from the hospital’s administrative database. Descriptive analyses were performed, with continuous variables reported as medians and interquartile ranges (IQR), and categorical variables as frequencies and proportions.

## 3. Results

**Literature review.** A total of 15 published articles, documenting 64 episodes of rare yeast fungaemia in Greece between 1999 and 2022, were identified. The data were derived from single-centre investigations carried out predominantly (93%, 14/15) in public tertiary care hospitals located in major urban centres, including Athens, Crete, Patras, Ptolemaida and Thessaloniki. Specifically, 10 studies detailed individual clinical cases [12,13,14,15,16,17,18,19,20,21], while two studies described small case series involving two patients each, one focusing on immunocompetent hosts [22] and the other on patients with COVID-19 [23]. Furthermore, three studies provided surveillance data on fungal BSIs, primarily focusing on species distribution and antifungal susceptibility patterns. These encompassed general hospital populations in Athens (*n* = 25) [24] and Patras (*n* = 11) [25], as well as a specialized neonatal and pediatric cohort (*n* = 14) [26] (Supplementary Table S1).

(i) **Patients’ characteristics**. Demographic data were available for the majority of the cases, with age reported in 39/64 episodes (61%) and sex in 38/64 episodes (59%). Among these patients, 63% (24/38) were male, with a median (range, IQR) age of 21 (0.03–76, 63) years. In particular, stratification by age group revealed that almost half of the episodes (46%, 18/39) occurred in pediatric patients, including 1 neonate (aged <28 days) and 8 infants (aged ≤1 year), while 18% (7/39) occurred in adults aged 18–59 years and 36% (14/39) in elderly individuals aged ≥60 years.

A significant proportion of patients had relevant underlying comorbidities, with hematological malignancies documented in 33% (13/39) and solid organ tumours in 15% (6/39) in both children and adults. Detailed data on risk factors were available for a more limited subset of patients (*n* = 14), providing insight into predisposing conditions for rare yeast fungaemia. All patients in this subgroup (100%, 14/14) had recently received broad-spectrum antibiotic therapy and the vast majority (93%, 13/14) had a history of venous catheterization, predominantly via central lines (11/14). Concomitant Gram-negative bacteraemia was reported in 50% (7/14), immunosuppressive states (including neutropenia) in 43% (6/14), and parenteral nutrition in 21% (3/14). Notably, all three patients with *Saccharomyces cerevisiae* BSIs and available exposure data had received *S. cerevisiae* var. *boulardii* as a probiotic therapy prior to the onset of fungaemia [13,23] (Table 1 and Table S1).

(ii) **Species**. The most frequently isolated causative pathogen was *S. cerevisiae*, accounting for 31% (20/64) of episodes, followed by *Rhodotorula mucilaginosa* (formerly *R. rubra*) in 30% (19/64), *Trichosporon asahii* in 25% (16/64), *Malassezia furfur* in 6% (4/64), *Magnusiomyces capitatus* (previously known by various synonyms including *Saprochaete capitata*, *Geotrichum capitatum*, *Blastoschizomyces capitatus*, *Trichosporon capitatum*, or *Dipodascus capitatus*) in 3% (2/64), *R. glutinis* in 3% (2/64) and *Moesziomyces aphidis* (formerly *Pseudozyma aphidis*) in 2% (1/64) (Table 1 and Table S1). Species identification methods were reported for 38/64 (59%) isolates and comprised MALDI-TOF MS in 66% (25/38), conventional phenotypic and biochemical approaches in 26% (10/38), and molecular amplification with sequencing of the ITS region in 8% (3/38) (Supplementary Table S1).

(iii) **Antifungal susceptibility.** Susceptibility testing was conducted in 58% (37/64) of reported cases, predominantly using gradient diffusion strips (78%, 29/37), while other commercial and reference broth microdilution methods were each applied in 11% (4/37) of tested isolates (Supplementary Table S1).

*S. cerevisiae* showed variable MICs for 5-flucytosine and most azoles, whereas fluconazole constantly exhibited high MIC values. Comparatively lower MICs were observed for amphotericin B and echinocandins. *Rhodotorula* spp. exhibited high MICs to echinocandins and moderate MICs to azoles and amphotericin B. Species-specific differences were recorded for 5-flucytosine and fluconazole, with 5-flucytosine demonstrating no activity and fluconazole moderate activity against *R. glutinis*, while the opposite was observed for *R. mucilaginosa*. *T. asahii*, *M. furfur*, *M. capitatus* and *M. aphidis,* which had diverse MIC patterns, characterized by elevated echinocandin MICs, variable 5-flucytosine and azole MICs, and reduced MICs to amphotericin B and selected azoles.

As gradient diffusion strips were calibrated based on the Clinical and Laboratory Standards Institute (CLSI) method, MICs were interpreted using CLSI ECVs for *S. cerevisiae* and amphotericin B (2 mg/L), anidulafungin (1 mg/L), micafungin (0.5 mg/L), caspofungin (2 mg/L), itraconazole (2 mg/L), fluconazole (32 mg/L), posaconazole (2 mg/L) and voriconazole (0.5 mg/L); for *R. mucilaginosa* and amphotericin B (2 mg/L), itraconazole (4 mg/L), posaconazole (4 mg/L) and voriconazole (16 mg/L); and for *T. asahii* and amphotericin B (1 mg/L), itraconazole (1 mg/L), fluconazole (8 mg/L) and posaconazole (1 mg/L) [27]. Overall, the proportions of non-wild type (WT) *S. cerevisiae*, *R. mucilaginosa* and *T. asahii* isolates were as follows: amphotericin B 0%, 0% and 30%; fluconazole 12%, 100% and 10%; voriconazole 0%, 100% and not determined (no established CLSI epidemiological cut-off values (ECVs); all isolates exhibited MICs ≤ 0.125 mg/L); posaconazole 38%, 0% and 0%; itraconazole 75%, 0% and 30%; and echinocandins 0%, 100% and 100%, respectively. The rates of *S. cerevisiae*, *R. mucilaginosa* and *T. asahii* isolates showing elevated 5-flucytosine MICs (> 0.5 mg/L) were 12%, 0% and 70%, respectively (Table 2 and Table S1).

(iv) **Antifungal therapy and outcome.** Data on antifungal treatment were available for a small proportion of patients (39%, 25/64). Breakthrough infections were documented in 8/25 episodes (32%), occurring predominantly (88%, 7/8) during administration of posaconazole (4/7) or fluconazole (3/7). Most cases (4/7) involved *R. mucilaginosa*, followed by *T. asahii* (2/7) and *M. capitatus* (1/7). The remaining *T. asahii*-related fungaemia was recorded in a patient who had been receiving anidulafungin for 17 days.

Amphotericin B was the most frequently administered agent among survivors. One patient with *T. asahii* fungaemia survived following treatment with conventional amphotericin B, escalated from 0.25 mg/kg/d to 1 mg/kg/d, for 21 days. Favourable outcomes were also reported in six patients who received liposomal amphotericin B: one with *M. aphidis* (7 mg/kg/d for 31 days), three with *R. mucilaginosa* (including one receiving 3 mg/kg/d for 10 days, while treatment details were not available for the remaining two) and one with *S. cerevisiae* (dose and duration not specified). Regarding azole therapy, both reported cases of *R. glutinis* fungaemia responded favourably to fluconazole, as did six *T. asahii* infections treated with voriconazole, although specific details for treatment were not provided. Echinocandins were used in three patients with *S. cerevisiae* infections: one received micafungin (14 days), whereas the other two underwent sequential treatment with anidulafungin (10 days) followed by fluconazole (14 days); all three survived, though dosing data were lacking. Notably, one *R. mucilaginosa* infection resolved without antifungal intervention, coinciding with the recovery from short-term neutropenia and mucositis.

On the other hand, one patient with *R. mucilaginosa* fungaemia received conventional amphotericin B, escalated from 5 mg/d to 0.6 mg/kg/d, for an unspecified duration and died. In a case of *M. capitatus*, liposomal amphotericin B (4.5 mg/kg/d) was initiated but discontinued after 4 days due to clinical deterioration, resulting in death. Another patient with a *T. asahii* infection succumbed after sequential treatment with liposomal amphotericin B (3.3 mg/kg/d for 5 days), followed by combination therapy with the same dose plus voriconazole (200 mg twice daily) for 10 additional days. A second patient with a *T. asahii* infection also died after combination therapy with liposomal amphotericin and voriconazole, although treatment details were not reported. Moreover, three patients died before a positive blood culture result was obtained (one each with *R. mucilaginosa*, *S. cerevisiae* and *M. capitatus*).

Taken together, *S. cerevisiae*, *R. mucilaginosa* and *T. asahii* infections were usually treated successfully with echinocandins, liposomal amphotericin B and voriconazole, respectively. The overall crude mortality rate was 28% (7/25); however, among patients who received antifungal therapy, mortality decreased to 19% (4/21) (Table 1 and Table S1).

**Single-centre experience from Attikon University General Hospital.** Over a 15-year period from 2010 to 2024, Attikon recorded a total of 670,875 hospital admissions, with an average of 44,725 admissions per year. During this time, 990 episodes of fungaemia were identified, the vast majority caused by *Candida* spp. (95%, 944/990).

(i) **Incidence**. A total of 29 episodes were attributed to rare yeasts, corresponding to an overall incidence rate of 3% (range: 0–9%). The estimated incidence density was 0.04 (range: 0–0.11) episodes per 1000 hospital admissions and 0.09 (range: 0–0.24) episodes per 10,000 patient days. Most episodes (76%, 22/29) occurred in patients admitted in internal medicine wards; surgical wards accounted for 17% (5/29) and ICUs for 7% (2/29). Rare yeast fungaemias were sporadically distributed throughout the study period, with no evidence of temporal or spatial clustering. The annual distribution was as follows: 1 case each in 2010, 2012 and 2020, 2 cases each in 2013, 2014 and 2015, 3 cases each in 2011 and 2012, 4 cases in 2017, and 5 cases each in 2018 and 2024 (Supplementary Table S2).
(ii) **Patients’ characteristics.** Within the cohort, 62% (18/29) of patients were male, with a median (range, IQR) age of 69 (17–88, 18) years. Hematological malignancies and solid organ tumours were the most prevalent underlying conditions, accounting for 31% (9/29) and 24% (7/29) of cases, respectively. At diagnosis, all patients were febrile, and 62% (18/29) were neutropenic or immunosuppressed. Concomitant Gram-negative bacteraemia was recorded in two cases and mixed fungaemia with *C. parapsilosis* was observed in one case. Detailed information on predisposing factors was available for 73% (24/29) of patients, all of whom were receiving antibiotic therapy and had central venous catheters (CVCs). Additional common risk factors included parenteral nutrition (58%, 14/24), diabetes mellitus (29%, 7/24), invasive surgical procedures (21%, 5/24, of which 3/5 were abdominal) and prolonged ICU stay (12%, 3/24). Among patients with *S. cerevisiae* fungaemia, for whom relevant data were available, 62% (5/8) had been receiving probiotics containing *S. cerevisiae* var. *boulardii* (Table 1 and Table S2). The median (range, IQR) time from hospital admission to onset of the infection was 27 (5–152, 26) days.
(iii) **Biomarkers**. Excluding patients with concomitant bacteraemia and candidaemia (*n* = 3), the median (range, IQR) C-reactive protein (CRP) level at the time of rare yeast-positive blood culture sampling was 88 (3–327, 85) mg/L, with most (92%) having > 10 mg/L, except two patients with 3.02–6.03 mg/L, both with *R. mucilaginosa* infection. Procalcitonin (PCT) values, available for 8/26 (31%) patients, showed a median (range, IQR) of 0.67 (0.13–1.27, 0.50) ng/mL, with most (62%) having > 0.5 ng/mL (three patients with *S. cerevisiae* infection and two patients with *R. mucilaginosa*), except three patients with 0.13 ng/mL (*T. asahii*), 0.29 ng/mL (*T. asahii*), and 0.45 ng/mL (*R. mucilaginosa*).

1,3-β-D-glucan (BDG) testing (Fungitell^®^ assay, Associates of Cape Cod, East Falmouth, MA, USA) was performed within ± 1 day of rare yeast-positive blood culture collection in two patients: one with *S. cerevisiae* (survived) and one with *T. asahii* (died). Both tests were negative, with values below the assay’s quantitative range (<31 pg/mL); no follow-up testing was conducted.

(iv) **Species**. The median (range, IQR) time to blood culture positivity was 4 (1–8, 3) days. The most frequently isolated species were *R. mucilaginosa* (41%, 12/29), *S. cerevisiae* (31%, 9/29) and *T. asahii* (21%, 6/29), followed by single isolates of *Apiotrichum loubieri* (formerly *Trichosporon loubieri*) and *M. capitatus* (3.5% each) (Table 1 and Table S2). Confirmatory species identification and AFST were successfully performed for all isolates except two *R. mucilaginosa* and two *S. cerevisiae*, which had not been stored. *M. capitatus* was the only isolate identified by ITS sequencing, as neither Vitek 2 (yielding inconclusive or unidentified results) nor MALDI-TOF MS (score value 1.84; genus-level identification) could assign species-level identification (Supplementary Table S2). The concordance between Vitek 2 and MALDI-TOF MS was 96% (24/25), with *A. loubieri* being the only species misidentified by Vitek 2 as *T. asahii* (identified with good confidence level).
(v) **Antifungal susceptibility.** *S. cerevisiae* exhibited low MICs for amphotericin B and echinocandins, and variable MICs for azoles, with voriconazole showing the highest in vitro activity. *R. mucilaginosa* displayed elevated MICs to echinocandins and fluconazole, moderate MICs to other azoles (voriconazole being the least active in vitro) and low MICs to amphotericin B. *T. asahii* exhibited high MICs to echinocandins and fluconazole, and moderate MICs to amphotericin B and other azoles, with voriconazole and isavuconazole demonstrating the highest in vitro activity.

Currently, official ECOFFs for rare yeasts have been published by EUCAST only for *S. cerevisiae* and amphotericin B (0.5 mg/L), anidulafungin (0.25 mg/L), micafungin (0.5 mg/L) and itraconazole (2 mg/L) [10]. As per EUCAST guidance on interpretation of MICs for rare yeast without breakpoints in breakpoint tables [11], amphotericin B may be considered a therapeutic option for *R. mucilaginosa* and *S. cerevisiae* isolates with MIC ≤ 1 mg/L, whereas *T. asahii* is considered intrinsically resistant. Fluconazole and voriconazole may be appropriate in selected clinical scenarios (e.g., non-severe infection, elevated-dose therapy, oral consolidation or when no better alternatives are available) for *S. cerevisiae* isolates with MIC ≤ 16 mg/L and ≤0.125 mg/L, respectively, but not for *R. mucilaginosa*, which is regarded as intrinsically resistant. Fluconazole may also be used in selected cases for *T. asahii* isolates with MIC ≤ 16 mg/L. Anidulafungin may be used as a therapeutic option for *S. cerevisiae* isolates with MIC ≤ 0.5 mg/L, but not for *R. mucilaginosa* or *T. asahii*, which are considered intrinsically resistant.

Thus, the % of non-WT/non-treatable isolates were 0% for *S. cerevisiae* and amphotericin B, anidulafungin, micafungin, fluconazole and voriconazole, *R. mucilaginosa* and amphotericin B, and *T. asahii* and fluconazole, whereas *R. mucilaginosa* was intrinsically resistant to echinocandins, fluconazole and voriconazole and *T. asahii* to amphotericin B and echinocandins. For the other drugs, median MICs were 0.5–1 mg/L for posaconazole and all three species, itraconazole and *R. mucilaginosa* and *T. asahii*, and 0.125–1 mg/L for isavuconazole and all three species. (Table 2 and Table S2). These values are higher than corresponding breakpoints for *C. albicans* (0.06 mg/L for itraconazole and posaconazole).

(vi) **Antifungal therapy**. Antifungal treatment data were available for 72% (21/29) of patients. At the time of fungaemia onset, one-third (33%, 7/21) were breakthrough infections occurring during treatment with anidulafungin (three patients; administered for 9–23 days), fluconazole (two patients; 11–25 days) and isavuconazole (two patients; 14–22 days). These infections were documented with *R. mucilaginosa* (*n* = 3; two during fluconazole and one during isavuconazole therapy), as well as in single cases of *M. capitatus*, *S. cerevisiae* and *T. asahii* (each during anidulafungin therapy), and *A. loubieri* (during isavuconazole therapy).

Antifungal treatment for the management of fungaemia was not administered in 4/21 (19%) cases due to death prior to blood culture results (two *S. cerevisiae*, one *R. mucilaginosa* and one *M. capitatus*). Of the 17 patients who received antifungal therapy, 10 were treated with monotherapy using liposomal amphotericin B (*n* = 4), an azole (*n* = 1) or an echinocadin (*n* = 5), while seven received sequential (*n* = 6) or concomitant (*n* = 1) combination therapy comprising liposomal amphotericin B + azole (*n* = 5), azole + echinocandin (*n* = 1) or liposomal amphotericin B + echninocandin (*n* = 1). In particular, three patients infected with *R. mucilaginosa* were treated with different regimens: short-course liposomal amphotericin B (5 mg/kg/d for 5 days), fluconazole (400 mg/d; duration unspecified), or sequential therapy comprising fluconazole (200 mg/d for 9 days) followed by liposomal amphotericin B (5 mg/kg/d for 7 days); all three patients died. Additionally, two patients with *S. cerevisiae* infections received either a short course of anidulafungin (100 mg/d for 6 days) or sequential therapy with fluconazole (400 mg/d for 6 days) followed by caspofungin (50 mg/d for 4 days), both of whom succumbed. Similarly, three patients with *T. asahii* infections died after treatment with distinct regimens: short-course anidulafungin (100 mg/d for 5 days), sequential therapy with liposomal amphotericin B (5 mg/kg/d for 3 days) followed by combination therapy with the same dose plus voriconazole (4 mg/kg twice daily) for 4 additional days, or liposomal amphotericin B (5 mg/kg/d) combined with voriconazole (4 mg/kg twice daily) for 17 days. The patient with the *A. loubieri* infection received a short course of liposomal amphotericin B (5 mg/kg/d for 4 days) and also died.

In contrast, four patients infected with *R. mucilaginosa* survived after prolonged treatment with liposomal amphotericin B (5 mg/kg/d), administered either as monotherapy (two cases; for 27 and 30 days) or as part of sequential therapy (two cases; for 19 and 20 days), following short courses of fluconazole (400 mg/d for 5 days) or anidulafungin (100 mg/d for 2 days). Likewise, two patients with *S. cerevisiae* infections survived after prolonged anidulafungin therapy (100 mg/d for 25 and 30 days), while another treated with caspofungin (50 mg/d; duration unspecified) also survived. One patient with *T. asahii* infection survived following sequential therapy with liposomal amphotericin B (5 mg/kg/d for 10 days) and voriconazole (4 mg/kg twice daily for 21 days).

Taken together, *R. mucilaginosa* fungaemia survival was observed only in patients who received at least 19 days of liposomal amphotericin B (alone or following short azole/echinocandin exposure), whereas all patients with shorter treatment durations succumbed to infection. Similarly, all survivors with *S. cerevisiae* infections received prolonged echinocandin therapy (≥25 days). On the other hand, all patients with *T. asahii* infections who received sequential or concomitant treatment with liposomal amphotericin B and voriconazole from 5 to 17 days died (Table 1 and Table S2).

(vii) **Mortality**. The median (range, IQR) duration of hospitalization following the collection of the rare yeast-positive blood culture was 17 (2–106, 22) days, while among patients who succumbed, the median (range, IQR) time from culture collection to death was 11 (2–106, 12) days. The overall crude mortality rate was 62% (18/29), comprising 61% (11/18) in the neutropenic/immunosuppressed subgroup and 64% (7/11) in the non-neutropenic/non-immunosuppressed subgroup. Among those who received antifungal therapy, mortality declined to 53% (9/17; 40% for *S. cerevisiae*, 43% for *R. mucilaginosa*, and 75% for *T. asahii*) (Table 1 and Table S2).

## 4. Discussion

Rare yeast fungaemia, although infrequent, represents a serious clinical concern. This study offers a comprehensive evaluation of infection patterns over a 15-year period within a general hospital setting. Rare yeast fungaemias accounted for 3% of all fungaemia episodes, occurring sporadically without evidence of outbreak clusters. The majority of infections were caused by *R. mucilaginosa*, *S. cerevisiae* and *T. asahii* (93% combined), primarily affecting patients with underlying hematological malignancies and solid organ tumours. All patients had CVCs and were receiving antibiotics. While most (62%) patients were neutropenic or otherwise immunocompromised, a significant proportion (38%) were neither neutropenic nor immunosuppressed. Notably, one-third of infections were classified as breakthrough, most commonly occurring during azole or echinocandin therapy, and diagnosis was established post-mortem in 19% of cases. Overall crude mortality was high at 62%, decreasing to 53% among those treated with antifungals.

The increasing clinical relevance of rare yeasts, traditionally regarded as non-pathogenic environmental saprophytes, reflects a shift in the landscape of invasive fungal infections. Although these fungi are ubiquitous in nature and commonly colonize human skin and mucosal surfaces, often forming part of the normal microbiota of the skin, gastrointestinal, respiratory and genitourinary tracts, they are now increasingly recognized as opportunistic pathogens [2]. Their emergence as causative agents of severe infections, such as fungaemia, is likely multifactorial. Contributing factors include the widespread and prolonged use of antifungal agents, the presence of indwelling medical devices, advances in oncologic and immunosuppressive therapies, as well as the extended survival of patients with complex comorbidities. In addition, recent advancements in diagnostic methodologies, particularly molecular techniques, have enhanced the detection and accurate identification of these uncommonly encountered yeasts, thereby reshaping our understanding of their distribution and clinical impact in contemporary settings [4]. Nevertheless, the epidemiology of rare yeast fungaemia is highly heterogeneous, not only between different countries but also within individual regions [2,4], highlighting the necessity for localized surveillance to identify key epidemiological patterns.

Accurate estimation of rare yeast fungaemia incidence is hindered by the scarcity of comprehensive, longitudinal epidemiological data, as most published studies span only a single year and are conducted at different times and under heterogeneous clinical settings. In Greece, our literature review identified only isolated case reports alongside three surveillance studies, the latter primarily addressing species distribution and antifungal susceptibility, over a 24-year span (1999–2022), precluding reliable national incidence estimates. At our institution, the incidence of rare yeast fungemia was 0.09/10,000 patient days accounted for 3% of all positive fungal blood cultures over a 15-year period, a proportion comparable to single-centre reports from general patient populations in Spain (2.4%) [28], Portugal (3.4%) [29] and Turkey (4.1%) [30], and consistent with the only available Greek data from a 4-year study in Patras (Northwestern Greece; 2.8%) [25]. Larger multicentre surveillance studies have reported lower rates of 0.9–2.0% across Asia [31], Europe [32,33,34] and Latin America [35], whereas higher rates have been documented in high-risk groups such as patients with malignancies (2.6–6.0%, 2.1/10,000 patient days) [36,37,38].

Although uncommon, rare yeast fungaemia carries significant clinical implications, particularly in immunocompromised patients. This is reflected in the predominance of cases within our cohort, as well as in the reviewed Greek literature and global data, where malignancies, especially hematological, are consistently identified as major risk factors [39,40,41,42]. However, we also observed a notable frequency of 38% in non-neutropenic/non-immunosuppressed individuals, consistent with previous reports [39,40,41,42]. In addition to immune dysregulation, other predisposing factors—mainly related to broad-spectrum antibiotic use, CVCs, previous antifungal therapy, recovery from invasive surgery, severe baseline diseases and parenteral nutrition—may also contribute to disease development [39,40,41]. All of our patients had CVCs and were on antibiotics, with a significant proportion also having a history of major surgical interventions (21%), prior antifungal exposure (33%) and parenteral nutrition (58%). Furthermore, the use of *S. cerevisiae* var. *boulardii* probiotics has emerged as a specific risk factor for *S. cerevisiae* fungaemia, particularly in critically ill hospitalized patients. A recent systematic review of 108 cases reported that 68% of *Saccharomyces* fungaemia episodes were associated with *S. cerevisiae* var. *boulardii* probiotic administration. Frequent accompanying risk factors included ICU stay (32%), total parenteral nutrition or enteral feeding (30%), gastrointestinal symptoms such as diarrhea (21%) and diabetes mellitus (13%). The overall all-cause mortality in the cohort was 36%, with no significant difference observed between probiotic-exposed and non-exposed patients (29% versus 9%), highlighting the clinical severity of these infections [39]. Notably, 62% of our patients with *S. cerevisiae*-related BSIs were receiving probiotics. These observations emphasize the importance of vigilant and individualized risk assessments that extend beyond traditionally immunocompromised individuals, in order to identify and mitigate infection-predisposing conditions. Furthermore, the median time from hospitalization to rare yeast fungaemia onset in our cohort was 27 days, which aligns with previously reported intervals ranging between 16 and 47 days [43,44].

Blood culture remains the cornerstone for diagnosing rare yeast BSIs, as it allows definitive identification of the causative pathogen and enables AFST. However, it is limited by low sensitivity and a slow turnaround time. In our cohort, fungaemia was diagnosed post-mortem in 19% of cases, and the median (IQR) time to blood culture positivity was 4 (3) days, consistent with previous reports of 4 to 5 (2–3) days [30,43]. The lack of surrogate fungal biomarkers [4], coupled with often non-specific clinical presentations that overlap with candidaemia, further complicates early recognition. These diagnostic delays may impede the timely initiation of appropriate targeted therapy, particularly in settings where empirical antifungal regimens do not cover intrinsically resistant rare yeasts, underscoring the need for faster, non-culture-based diagnostic tools.

The non-invasive nature and rapid turnaround time of commonly used inflammatory markers render them valuable adjuncts in the early detection of BSIs. Excluding patients with concomitant bacteraemia or other fungaemia, CRP levels in our cohort (median 88 mg/L) were comparable to those observed in febrile patients with documented fungaemia (mean 113 ± 69 mg/L) [45], suggesting a potential role for CRP in the inflammatory response to rare yeast fungaemia. The sensitivity and specificity of CRP at the cutoff value of 85.75 mg/L was 66.7% and 71.1%, respectively [45]. In contrast, PCT levels (median 0.67 ng/mL; available for 31% of patients) were nearly half of those observed in the same patient population (mean 1.04 ± 9.81 ng/mL) [45]. This difference may reflect the lower endotoxin-like activity of rare yeasts compared to common fungal pathogens and warrants further investigation to clarify the role of PCT in differentiating rare yeast BSIs from candidaemia. The sensitivity and specificity of PCT at the cutoff value of 0.3035 ng/mL was 88.9% and 47.2%, respectively [45].

Regarding established serological fungal biomarkers, international guidelines provide only marginal support for the use of serum BDG testing as a screening tool [2]. While it may aid in detecting some cases, such as fungaemia due to *Trichosporon* spp., *M. capitatus* and *M. clavatus*, with reported sensitivities of 48–82% [41,46], 64% and 43% [47], respectively, there is currently insufficient evidence to support its routine use as a standalone diagnostic test in rare yeast BSIs, whereas a negative result does not exclude infection [2]. In our cohort, BDG testing was performed in 2/29 patients, one with *S. cerevisiae* and one with *T. asahii*, and both tests returned negative. Of note, *T. asahii* and *T. mucoides* share with *Cryptococcus* spp. the ability to produce glucuronoxylomannan, and prior studies have suggested that serum glucuronoxylomannan may be more suitable than BDG for the diagnosis of *Trichosporon* fungaemia [48]. This polysaccharide antigen has been detected by cryptococcal antigen latex agglutination assays in 27–54% of sera from patients with disseminated trichosporonosis [41,49]. Therefore, international guidelines advise caution when interpreting cryptococcal antigen tests in blood, as cross-reactivity may occur in the setting of *Trichosporon* infection [2].

Molecular assays performed directly on positive blood cultures have been associated with earlier initiation of targeted therapy, shorter hospitalization and improved outcomes. However, genus-/species-specific serum PCR assays for rare yeasts remain scarce and insufficiently validated in clinical settings [49,50,51]. PCR assays targeting a wide spectrum of fungal pathogens may offer a more practical alternative, provided they allow at least genus-level identification, since detection without taxonomic resolution limits clinical utility, increases false-positive risk and delays appropriate treatment. Species-level identification could also be valuable in certain cases, as species-specific differences in antifungal susceptibility and therapeutic response have been reported. For instance, *R. glutinis* fungaemias have shown survival with fluconazole treatment (MIC 2 mg/L) [22], as opposed to *R. mucilaginosa* infections which involve pathogens considered intrinsically fluconazole-resistant (MIC_90_ > 64 mg/L) [2]. Moreover, when identification requires downstream sequencing, as is the case with pan-fungal PCR assays, the diagnostic delay may substantially diminish clinical benefit. The GenMark ePlex Fungal Pathogen Panel is currently the only FDA-approved molecular blood culture identification panel, which enables rapid detection of 15 fungal targets, including *Rhodotorula* spp., with high sensitivity (96.2%) and specificity (99.9%) for the species [52]. Nevertheless, real-world data have revealed a higher false-positive rate (up to 75%) for *Rhodotorula*, especially when co-detected with *Candida* spp., warranting cautious interpretation of such results to avoid unnecessary exposure to antifungals [53]. Other molecular tools (e.g., oligonucleotide-based arrays) remain under-evaluated [54]. Further studies are needed to validate molecular diagnostics for rare yeast BSIs, particularly in the context of breakthrough infections, before such technologies can be widely adopted.

Considerable variability in the distribution of causative agents of rare yeast fungaemia has been noted at both international and sub-national levels [2,5]. *R. mucilaginosa* predominated in our cohort (41%), mirroring findings in general patient populations from Italy (41%) [55], Portugal (75%) [29] and Spain (42–54%) [28,56], but contrasting with the Greek study from Patras, where it ranked second alongside *S. cerevisiae* (18% each) [25]. This geographical concordance with other Mediterranean countries potentially reflects shared environmental and climatic conditions, as well as comparable healthcare practices, as previously described for candidaemia [24]. Notably, among rare yeasts, *Rhodotorula* spp. were the most frequently encountered contaminants in the hospital environment [57,58], with a higher isolation frequency from the air than from surfaces [59]. Since species of this genus are part of the human mycobiome, particularly of the gastrointestinal tract and oral/nasal cavity [60], they may originate from both patients and healthcare workers, contributing to indoor air contamination, similar to what has been described for commensal bacteria, which can become aerosolized from the skin and oral mucosa [61]. Conversely, intranational heterogeneity could be attributed to regional differences in environmental reservoirs, patient populations, clinical management protocols and diagnostic capabilities, highlighting the complex determinants that shape fungal epidemiology. *S. cerevisiae*, which, like *Rhodotorula* spp., exhibits biofilm-forming capacity, was the second most frequent rare yeast (31%), whereas it was the leading species in previous population-based studies conducted in Belgium (88%) [62] and Denmark (65%) [32]. *Trichosporon* spp. (including *Apiotrichum* spp. according to the updated nomenclature) ranked third in our cohort (24%; 86% *T. asahii*), with these fungi predominantly found in tropical and temperate regions, as evidenced by their distribution as the primary cause of rare yeast fungaemia in large-scale studies performed in Argentina (44%) [35], Australia (44%) [63] and several Asian countries (52%) [31]. Interestingly, *T. asahii* was also the predominant species in the study from Patras (54%) [25]. Lastly, a single case of *M. capitatus*-related fungaemia was identified (3%), marking the third documented case in Greece based on our literature review [14,17], with this yeast emerging as a cause of invasive infections, particularly in profoundly immunosuppressed individuals, and most frequently reported in the Mediterranean basin and Central Europe [42,64,65].

International guidelines highlight the importance of accurate identification of rare yeasts isolated from sterile sites, favouring molecular techniques, such as sequencing, and/or MALDI-TOF MS over conventional approaches [2], whose accuracy is suboptimal beyond certain rare yeast species [66]. While genus-level identification may be sufficient to guide treatment in selected cases, species-level identification is strongly recommended for epidemiological surveillance [2]. MALDI-TOF MS has gradually become a cornerstone in fungal diagnostics, allowing direct detection of arthroconidial rare yeasts from positive blood cultures with reported confidence levels of 80–99.9% [67], while all currently available databases have performed equally well for species-level identification of uncommon yeasts [68]. However, its availability remains resource-dependent and restricted access may contribute to misidentification and underestimation of rare yeast infections. At the same time, the enduring value of classical microbiological methods should not be overlooked, particularly in resource-limited settings. For instance, direct Gram-stain examination of positive blood cultures can support early presumptive identification of arthroconidial rare yeasts, aiding in the avoidance of empirical echinocandin use, to which these organisms are intrinsically resistant [69].

As regards AFST, current guidelines recommend MIC testing using reference broth microdilution methods for rare yeasts [2]. Indeed, commercial AFST assays, optimized for common *Candida* spp., may have limited applicability to new species with distinct characteristics (e.g., growth rate, metabolic status). For example, the agreement between azole MICs obtained by CLSI broth microdilution and Etest methods for rare yeasts was only moderate (53–84%) [70]. Hence, while widely used in routine practice due to their user-friendliness, including in Greece as noted in our literature review (89% of MIC data), their results should be interpreted with caution. However, broth microdilution MIC data for rare yeasts remain scarce, and since broth microdilution protocols were primarily developed for fast-growing yeasts, modifications may be required for some rare yeasts [12,41,42].

Our AFST results reinforce the well-documented pattern of antifungal resistance in rare yeast BSIs that complicates therapeutic choices, aligning with prior published EUCAST broth microdilution studies [7,41,42]. In particular, echinocandins were ineffective against *R. mucilaginosa*, *M. capitatus* and *T. asahii*, but remained active in vitro against *S. cerevisiae*. Similarly, elevated fluconazole MIC values were observed for all isolates tested. Amphotericin B demonstrated consistently good in vitro activity against rare yeasts, while the efficacy of other azoles was species-dependent, with voriconazole displaying potent activity against *S. cerevisiae* and *T. asahii*. Although EUCAST provides guidance for MIC interpretation in the absence of species-specific clinical breakpoints [11], the lack of established breakpoints limits precise clinical interpretation of AFST results [5,7]. In fact, AFST for rare yeasts remains essential for enriching epidemiological knowledge, and occasionally for guiding treatment in therapeutic failures (*Rhodotorula* infections), as well as monitoring the emergence of non-WT isolates (trichosporonosis) [2]. Despite limited direct evidence linking susceptibility data to clinical outcomes, these results are valuable for surveillance and the establishment of ECVs/ECOFFs for these uncommon yeasts. Collectively, our findings further support the importance of basing treatment selection on species-level identification and AFST of the etiological agents [71] as there is no “one-size-fits-all” approach, while also bringing to the forefront the need for robust susceptibility data reporting.

Given the above, breakthrough rare yeast fungaemias remain difficult to predict and manage. Antifungal prophylaxis, despite its preventative intent, may inadvertently promote the selection of intrinsically resistant organisms. Likewise, empirical regimens based on fluconazole, and particularly those involving echinocandins, which are now widely adopted as first-line agents in high-risk settings, may require careful reassessment in patients with prior antifungal exposure to prevent therapeutic failure. Physicians should bear in mind that yeast fungaemia does not always mean *Candida*, especially in cases of breakthrough fungaemia in severely immunocompromised patients [72]. In our cohort, breakthrough BSIs occurred in one-third of patients, a rate consistent with both our national review (32%) and international reports (mean (range) 40% (30–50%)) [33,43,56]. Higher rates (49–56%) have been described in cohorts with patients with malignancies [37,38].

Notably, 5/7 breakthrough episodes involved species intrinsically resistant to the antifungal administered (anidulafungin, fluconazole or isavuconazole), as previously described [43,56,73,74]. Exceptions included one *S. cerevisiae* isolate during anidulafungin therapy (MIC 0.06 mg/L) and the single *A. loubieri* isolate during isavuconazole therapy (MIC 0.03 mg/L), although isavuconazole is not approved for the treatment of invasive yeast infections. Similar cases of echinocandin-breakthrough *S. cerevisiae* fungaemia and isavuconazole-breakthrough trichosporonosis have been described in the literature [75,76]. The diversity of breakthrough pathogens across different antifungal classes suggests distinct resistance mechanisms among rare yeasts, reinforcing the need for ongoing surveillance and development of optimized, evidence-based treatment strategies. These considerations are particularly relevant in local contexts such as Greece, where mould-active prophylaxis is widely implemented (95%) among hematology patients [77], and where the use of echinocandin has markedly increased in recent years, while fluconazole consumption has remained consistently high over time [78]. Our findings, therefore, highlight the importance of sustained epidemiological monitoring, antifungal stewardship and the development of centre-specific management algorithms tailored to regional resistance patterns and institutional clinical expertise.

According to international guidelines, liposomal amphotericin B, either as monotherapy or combined with 5-flucytosine, is recommended for the treatment of *R. mucilaginosa* fungaemia, with a strongly endorsed course of 2–3 weeks. Voriconazole serves as the first-line agent for invasive trichosporonosis, typically administered for 2 weeks in the absence of dissemination from fungaemia with organ involvement. In systemic infections caused by *S. cerevisiae*, empirical use of all major antifungal classes may be appropriate; however, definitive therapy should be guided by AFST results obtained via the reference broth microdilution method and adjusted according to the clinician’s assessment of the specific clinical context. For BSIs, treatment should be maintained for at least 2 weeks following the last negative blood culture, guided by clinical response [2]. In our cohort, patients who survived generally received prolonged (≥19 days) antifungal regimens tailored to the pathogen: liposomal amphotericin B for *R. mucilaginosa*, voriconazole for *T. asahii* and echinocandins for *S. cerevisiae*. In contrast, patients who succumbed either underwent short courses of therapy (≤7 days); received treatments that did not align with, or were poorly supported by, international recommendations [2], including fluconazole for *R. mucilaginosa* (two cases), combination therapy with azoles and polyenes in non-refractory cases or anidulafungin for *T. asahii* (three cases); or died before treatment initiation (four cases). These findings are a matter of concern underscoring the critical importance of timely diagnosis, prompt initiation of empirical antifungal therapy in at-risk populations pending species identification and susceptibility profiling based on local epidemiological patterns, and adequate duration of targeted antifungal treatment.

Despite the relatively low virulence of some rare yeasts, delays and challenges in diagnosis and treatment contribute significantly to the high mortality rates observed in rare yeast fungaemia. In our cohort the overall crude rate was 62%, which aligns with global estimates for the general patient population, ranging from 24% to 60% (mean 47%) [30,33,35,44,56,63,71], though it is notably higher than the 18% reported in the Greek study from Patras [25]. The variability in mortality rates across different studies may reflect differences in patient characteristics, antifungal treatment strategies and diagnostic timelines; however, the consistently high mortality underscores the need for enhanced diagnostic approaches and more targeted treatment protocols. Interestingly, both neutropenic/immunosuppressed and non-neutropenic/non-immunosuppressed patients exhibited similarly elevated rates (61% versus 64%), which is consistent with earlier reports of a mean (range) mortality of 46% (34–89%) in high-risk patients, such as those with cancer, hematological malignancies and immune disorders [38,79,80,81]. This suggests that factors beyond host immunity, such as delayed clinical recognition, inadequate empirical therapy or suboptimal catheter/source control, may significantly contribute to poor outcomes, even among immunocompetent yet severely ill individuals or those with miscellaneous underlying conditions predisposed to rare yeast fungaemia.

Although antifungal therapy was administered to the majority of our patients, it did not significantly reduce crude mortality, which remained at 53%. Focusing on specific pathogens, 51% of patients with *Trichosporon* spp.-related BSIs experienced a fatal outcome [41], a rate close to our findings (75%). Similarly, the all-cause mortality rate associated with *S. cerevisiae*-induced fungaemia was 36% [39], corroborating our results (40%). Nonetheless, an overall mortality rate of 13% for *Rhodotorula* spp. BSIs has been reported [40], nearly three times lower than the 43% observed in our cohort. This discrepancy could be attributed to differences in patient risk profiles, clinical management and institutional protocols. Furthermore, the median (IQR) time-to-death of 11 (12) days following blood culture positivity is consistent with a previous study reporting 10 (19) days [43], reflecting the often rapid and severe clinical course of rare yeast fungaemia.

This study has several limitations that warrant consideration. The retrospective nature of the study resulted in incomplete clinical data for some cases, along with insufficient information on the extent of organ involvement, catheter management (despite strong recommendations for catheter removal) and infection-related mortality. In the absence of standardized diagnostic criteria, it cannot be ruled out that some cases may have represented pseudofungaemia; however, all isolates were deemed clinically relevant by the attending clinicians. Despite the variability in blood culture contamination rates (0.6–12.5%) [82], the minimal contribution of rare yeasts supports their clinical relevance upon detection. Lastly, the relatively small number of cases constrained the ability to conduct comprehensive statistical analyses on predisposing factors and outcomes. While this limitation is inherently linked to the rarity of rare yeast fungaemia, it nonetheless restricts the strength of conclusions related to optimal therapeutic strategies and prognostic indicators.

Despite these challenges, this study represents a comprehensive assessment of rare yeast fungaemia in a Greek general hospital setting, providing valuable and contemporary insights into its epidemiology, clinical features, microbiology and management. As such, it contributes to a better understanding of an infection that has hitherto been relatively underreported in the local context and may serve as a benchmark for future surveillance efforts. Strengthening regional surveillance will help delineate the true burden of disease, identify local unmet needs, and support more informed diagnostic and therapeutic strategies. Moreover, it supplements existing epidemiological data on invasive rare yeast infections in Greece by presenting additional cases not included [12,13,14,21,23,25,26] in the latest guidelines [2].

## 5. Conclusions

This thorough epidemiological report on rare yeast fungaemia in Greece underscores the frequent occurrence of breakthrough infections and the diagnostic and therapeutic obstacles it poses, highlighting the importance of prompt recognition, even beyond classical settings of immunosuppression, and of evidence-based empirical treatment. Given the intrinsic resistance of rare yeast species to most licenced antifungals, increasing awareness of their local epidemiology is imperative. Future multicentre, population-based surveillance studies, both nationally and internationally, are warranted to better capture real-world incidence and elucidate clinical patterns and outcomes. The considerable mortality rates observed further reinforce the need for developing and approving novel agents, alongside optimizing therapeutic strategies tailored to these uncommon yet difficult-to-treat pathogens.

## Figures and Tables

**Table 1:** Demographic, clinical and microbiological features of rare yeast fungaemia cases in Greece based on literature review and in the present study in “Attikon” hospital.

		Literature Review	Attikon Hospital
**Number of cases**		64	29
Patients’ characteristics *			
**Sex**	Male	24/38 (63%)	18/29 (62%)
	Female	14/38 (37%)	11/29 (38%)
**Age** (years) (median (range, IQR))		21 (0.03–76, 63)	50 (18–83, 27)
**Underlying condition**	Hematological malignancy	13/39 (33%)	9/29 (31%)
	Solid organ tumour	6/39 (15%)	7/29 (24%)
**Other characteristics**	Neutropenia/Immunosuppression	6/14 (43%)	18/29 (62%)
	Concomitant Gram-negative bacteraemia	7/14 (50%)	2/29 (7%)
Risk factors *	Antibiotic therapy	14/14 (100%)	24/24 (100%)
	Central/Peripheral venous catheter	13/14 (93%)	24/24 (100%)
	Parenteral nutrition	3/14 (21%)	14/24 (58%)
	Diabetes mellitus	1/14 (7%)	7/24 (29%)
	Invasive surgical procedure	2/14 (14%)	5/24 (21%)
**Causative pathogen**	*R. mucilaginosa*	19/64 (30%)	12/29 (41%)
	*S. cerevisiae*	20/64 (31%)	9/29 (31%)
	*T. asahii*	16/64 (25%)	6/29 (21%)
	Other	9/64 (14%)	2/29 (7%)
**Breakthrough infection ^#^**	Total number	8/25 (32%)	7/21 (33%)
	AFG	1/8 (12%)	3/7 (42%)
	FLC	3/8 (38%)	2/7 (29%)
	ISA	-	2/7 (29%)
	PSC	4/8 (50%)	-
**Antifungal therapy ^#^**	LAMB	6/25 (24%)	4/21 (19%)
	AMB	2/25 (8%)	-
	Echinocandins	1/25 (4%)	5/21 (23.5%)
	Azole	8/25 (32%)	1/21 (5%)
	LAMB + azole ^^^	2/25 (8%)	5/21 (23.5%)
	LAMB + echinocandin ^^^	-	1/21 (5%)
	Azole + echinocandin ^^^	2/25 (8%)	1/21 (5%)
	No therapy	4/25 (16%)	4/21 (19%)
**Crude within hospital mortality ^#^**	Prior to blood culture results	3/25 (12%)	4/21 (19%)
	Among those who received antifungal therapy	4/21 (19%)	9/17 (53%)

* Among patients with available data. ^#^ Among patients with available treatment data. ^^^ Sequential or concomitant combination therapy. **Abbreviations.** AFG, anidulafungin; AMB, amphotericin B; FLC, fluconazole; ISA, isavuconazole; LAMB, liposomal amphotericin B; PSC, posaconazole.

**Table 2:** Antifungal susceptibility patterns of rare yeast isolates recovered from fungaemia cases in Greece based on literature review and in the present study in “Attikon” hospital.

	Literature Review (GDS/CLSI ECVs)			Attikon Hospital (EUCAST BMD/ECOFFs)		
	*R. mucilaginosa* (*n* = 4)	*S. cerevisiae* (*n* = 8)	*T. asahii* (*n* = 10)	*R. mucilaginosa* (*n* = 10)	*S. cerevisiae* (*n* = 7)	*T. asahii* (*n* = 6)
**Amphotericin B**						
Median (range) MIC (mg/L)	2 (0.5–2)	0.25 (0.016–0.5)	0.5 (0.06–> 32)	0.25 (0.25–0.5)	0.25 (0.25–0.5)	1 (1–1)
Number (%) of non-WT isolates	0 (0%)	0 (0%)	3 (30%)	[0 (0%)] ^b^	0 (0%)	- ^a^
**Anidulafungin**						
Median (range) MIC (mg/L)	>32 (>32–>32)	0.03 (0.008–0.125)	>32 (>32–>32)	>8 (4–> 8)	0.125 (0.06–0.125)	4 (4–>8)
Number (%) of non-WT isolates	- ^a^	0 (0%)	- ^a^	- ^a^	0 (0%)	- ^a^
**Micafungin**						
Median (range) MIC (mg/L)	>32 (>32–>32)	0.06 (0.016–0.25)	>32 (>32–>32)	>8 (4–>8)	0.125 (0.06–0.125)	>8 (4–>8)
Number (%) of non-WT isolates	- ^a^	0 (0%)	- ^a^	NA	0 (0%)	NA
**Caspofungin**						
Median (range) MIC (mg/L)	>32 (>32–>32)	0.25 (0.06–0.25)	>32 (>32–>32)	>8 (>8–>8)	0.5 (0.5–1)	>8 (4–>8)
Number (%) of non-WT isolates	- ^a^	0 (0%)	- ^a^	NA	NA	NA
**Fluconazole**						
Median (range) MIC (mg/L)	>256 (>256–>256)	16 (4–>256)	2 (1–16)	>64 (>64–>64)	16 (8–16)	16 (8–16)
Number (%) of non-WT isolates	- ^a^	1 (12%)	1 (10%)	- ^a^	(0 (0%)) ^b^	(0 (0%)) ^b^
**Voriconazole**						
Median (range) MIC (mg/L)	2 (1–2)	0.25 (0.03–0.5)	0.06 (0.03–0.125)	2 (1–4)	0.125 (0.125–0.125)	0.125 (0.06–0.25)
Number (%) of non-WT isolates	0 (0%)	0 (0%)	NA	- ^a^	(0 (0%)) ^b^	NA
**Posaconazole**						
Median (range) MIC (mg/L)	0.25 (0.25–0.5)	2 (1–8)	0.5 (0.25–1)	1 (0.5–2)	1 (0.25–1)	0.5 (0.25–0.5)
Number (%) of non-WT isolates	0 (0%)	3 (38%)	0 (0%)	NA	NA	NA
**Itraconazole**						
Median (range) MIC (mg/L)	1 (1–1)	4 (4–>32)	1 (0.5–4)	0.5 (0.5–1)	8 (0.5–-> 8)	0.5 (0.125–0.5)
Number (%) of non-WT isolates	0 (0%)	6 (75%)	3 (30%)	NA	4 (57%)	NA
**Isavuconazole**						
Median (range) MIC (mg/L)	0.5 (0.25–0.5)	0.125 (0.03–0.5)	0.125 (0.03–0.5)	0.5 (0.25–2)	1 (0.125–1)	0.125 (0.06–0.5)
Number (%) of non-WT isolates	NA	NA	NA	NA	NA	NA
**5-Flucytosine**						
Median (range) MIC (mg/L)	0.06 (0.03–0.06)	0.06 (0.008–1)	16 (0.25–>32)	NP	NP	NP
Number (%) of non-WT isolates	NA	NA	NA	NA	NA	NA

^a^ Intrinsic resistance. ^b^ There are no ECOFFs for these species and drugs. The number (%) corresponds to isolates with MICs higher than the MIC cutoffs proposed by EUCAST for MIC interpretation for rare yeasts without established breakpoints [11], >1 mg/L for amphotericin B, >16 mg/L for fluconazole and >0.125 mg/L for voriconazole. **Abbreviations.** BMD, broth microdilution; EUCAST, European committee on antimicrobial susceptibility testing; GDS, gradient diffusion strip; NA, not available; NP, not performed; WT, wild type.

## Data Availability

Data available on request. Accession numbers for sequences deposited in GenBank include OR400644.

## Supplementary Materials

The following supporting information can be downloaded at: https://www.mdpi.com/article/10.3390/jof12030187/s1, Table S1: Reported cases of bloodstream infection due to rare yeasts in Greece, presented chronologically; Table S2: Reported cases of fungaemia caused by rare yeasts in Attikon University General Hospital (2010–2024), presented chronologically.
