# Supplementary material for: Beyond Candida: Epidemiological Insights into Rare Yeast Fungaemia in Greece from a 15-Year Hospital-Based Study and Literature Review
Source: J Fungi (Basel). 2026 Mar 5;12(3):187. doi: 10.3390/jof12030187 (PMC13028254; doi:10.3390/jof12030187)
Supplement: Supplementary file 1 [file jof-12-00187-s001.zip › jof-4134236-supplementary.pdf]

Supplementary Table S1. Reported cases of bloodstream infection due to rare yeasts in Greece, presented chronologically.

No	Year of diagnosis (city)	Age (years) Sex	Underlying conditions and risk factors (immune status)	Pathogen (identification method)	Antifungal susceptibility (testing method)	Breakthrough infection (prior antifungal, duration)	Antifungal therapy	Outcome	Reference
1	1999 (Athens)	57 NA	Gastrointestinal malignancy, colectomy, PN, CVC, ABT (neutropenic)	*Rhodotorula mucilaginosa* (phenotypic and biochemical approaches)	FLC >64 mg/L, ITC 4 mg/L, AMB 0.5 mg/L, 5-FC ≤0.125 mg/L (CLSI BMD)	No	AMB (duration NA; increased from 5 mg/d to 0.6 mg/kg/d)	Death	[21]
2	2000 (Athens)	21 F	NH lymphoma, HSCT recipient, PN, CVC, ABT (neutropenic)	*Rhodotorula mucilaginosa* (API ID 32C)	FLC >256 mg/L, ITC 1 mg/L, AMB 0.25 mg/L, 5-FC 0.06 mg/L (Sensititre YeastOne)	Yes (FLC, 200 mg/d x 9 days)	LAMB (3 mg/kg/d x 10 days)	Survival	[20]
3	2001 (Crete)	76 M	Colonic malignancy, gastrointestinal mucositis, COPD, CTX, PVC, ABT (neutropenic)	*Rhodotorula mucilaginosa* (API 20C AUX)	NA	No	No	Survival (infection cleared spontaneously after recovery from neutropenia/mucositis)	[19]
4	2002 (Thessaloniki)	0.5 F	ELBW, RDS, CVC, ABT, Gram-negative BSI (non-neutropenic)	*Trichosporon asahii* (API ID 32C)	5-FC 8 mg/L, AMB/FLC 1 mg/L, ITC 0.5 mg/L (CLSI BMD)	No	AMB (21 days; 0.25 mg/kg/d escalated daily to 1 mg/kg/d)	Survival (negative cultures by day 4 of AMB)	[18]
5	2003 (Athens)	69 M	AML, CTX, CVC, ABT (neutropenic)	*Magnusiomyces capitatus* (API 32ID)	FLC 16 mg/L, 5-FC 2 mg/L, ITC 1 mg/L, AMB/CAS 0.5 mg/L, VRC 0.25 mg/L (CLSI BMD)	Yes (FLC, 400 mg/d x 5 days)	LAMB (4.5 mg/kg/d x 4 days)	Death	[17]
6	2004 (Thessaloniki)	13 M	ALL, CTX, CVC, ABT (neutropenic)	*Trichosporon asahii* (API ID 32C)	FLC 2 mg/L, AMB/ITC/PSC 0.25 mg/L, VRC ≤0.06 mg/L (CLSI BMD)	No	LAMB (3.3 mg/kg/d x 5 days) → LAMB (3.3 mg/kg/d) + VRC (200 mg bid) x 16 days	Death	[16]
7	2007 (Crete)	64 M	MRSA and Gram-negative BSI, PVC, ABT (non-neutropenic)	*Rhodotorula glutinis* (Vitek 2 YST)	5-FC >32 mg/L, FLC 2 mg/L (GDS)	No	FLC (duration NA; switched from 400 mg/d [duration NA] to po [dose NA] x 30 days)	Survival (negative cultures by day 2 of FLC)	[22]
8	2007 (Crete)	65 M	Community-acquired pneumonia, PVC, ABT (non-neutropenic)	*Rhodotorula glutinis* (Vitek 2 YST)	5-FC >32 mg/L, FLC 2 mg/L (GDS)	No	FLC (duration NA; switched from 400 mg/d [duration NA] to po [dose NA] x 30 days)	Survival	[22]
9	2012 (Patras)	48 F	Ovarian malignancy, bowel necrosis, abdominal surgeries, Gram-negative BSI, PN, CVC, ABT (immunosuppressed)	*Rhodotorula mucilaginosa* (Vitek 2 YST)	FLC >256 mg/L, AFG/MFG/CAS >32 mg/L, AMB/VRC 2 mg/L, ITC 1 mg/L, PSC 0.25 mg/L, 5-FC 0.06 mg/L (GDS)	Yes (FLC, NA)	No	Death*	[15]
10	2016 (Athens)	63 M	HL, ALS, gastrostomy, Gram-negative BSI, CVC, ABT (non-neutropenic)	*Magnusiomyces capitatus* (API 32C)	AFG 8 mg/L, FLC/MFG/CAS 4 mg/L, ITC 2 mg/L, PSC 0.5 mg/L, AMB/VRC 0.25 mg/L, 5-FC 0.06 mg/L (MICRONAUT-AM)	No	No	Death*	[24]
11-22	2009-2018 (Athens)	NA	NA	12 cases of *Rhodotorula mucilaginosa* (NA)	NA	NA	NA	NA	[14]
23-31	2009-2018 (Athens)	NA	NA	9 cases of *Saccharomyces cerevisiae* (NA)	NA	NA	NA	NA	[24]
32-35	2009-2018 (Athens)	NA	NA	4 cases of *Trichosporon asahii* (NA)	NA	NA	NA	NA	[24]
36	2019 (Patras)	2 M	Gastroenteritis, ABT, SBP (non-neutropenic)	*Saccharomyces cerevisiae* (NA)	NA	No	MFG (dose NA x 14 days)	Survival	[13]
37	2020 (Ptolemaida)	76 M	Acute RDS, COVID-19, Gram-negative BSI, haemodialysis, CVC, ABT, SBP (non-neutropenic)	*Saccharomyces cerevisiae* (ITS sequencing)	5-FC/FLC 4 mg/L, AMB 1 mg/L, ITC 0.5 mg/L, VRC 0.125 mg/L, AFG 0.06 mg/L, PSC 0.03 mg/L (ATB Fungus 3; AFG/PSC by GDS)	No	AFG (dose NA x 10 days) → FLC (dose NA x 14 days)	Survival	[23]

38	2020 (Ptolemaida)	73 M	Diabetes, acute RDS, COVID-19, Gram-negative BSI, haemodialysis, CVC, ABT, SBP (non-neutropenic)	*Saccharomyces cerevisiae* (ITS sequencing)	5-FC/FLC 4 mg/L, AMB/ITC 0.5 mg/L, VRC 0.125 mg/L, PSC 0.06 mg/L, AFG ≤0.002 mg/L (ATB Fungus 3; AFG/PSC by GDS)	No	AFG (dose NA x 7 days) → FLC (dose NA x 14 days)	Survival	[23]
39	2008-2020 (Athens)	13 M	AML, PN, detailed RF NA (NA)	*Malassezia furfur* (MALDI-TOF MS, Bruker)	FLC >256 mg/L, AFG/CAS/MFG/5-FC >32 mg/L, AMB 0.25 mg/L, ISA 0.125 mg/L, PSC 0.06 mg/L, VRC/ITC 0.03 mg/L (GDS)	NA	NA	NA	[26]
40	2008-2020 (Athens)	0.3 M	Prematurity, Hirschsprung disease, colostomy, detailed RF NA (NA)	*Malassezia furfur* (MALDI-TOF MS, Bruker)	AFG/CAS/MFG/5-FC/VRC >32 mg/L, FLC/ISA 1 mg/L, AMB 0.5 mg/L, ITC/PSC 0.125 mg/L (GDS)	NA	NA	NA	[26]
41	2008-2020 (Athens)	0.2 M	Congenital heart disease, duodenal atresia, detailed RF NA (NA)	*Malassezia furfur* (MALDI-TOF MS, Bruker)	FLC 64 mg/L, AFG/CAS/MFG/5-FC >32 mg/L, AMB/ITC/ISA 0.125 mg/L, VRC/PSC 0.06 mg/L (GDS)	NA	NA	NA	[26]
42	2008-2020 (Athens)	0.03 F	Necrotizing enterocolitis, surgical procedure, PN, detailed RF NA (NA)	*Malassezia furfur* (MALDI-TOF MS, Bruker)	AFG/CAS/MFG >32 mg/L, 5-FC 16 mg/L, ISA 0.5 mg/L, AMB/FLC 0.25 mg/L, VRC/ITC/PSC 0.125 mg/L (GDS)	NA	NA	NA	[26]
43	2008-2020 (Athens)	12 M	ALL, detailed RF NA (NA)	*Rhodotorula mucilaginosa* (MALDI-TOF MS, Bruker)	FLC >256 mg/L, AFG/CAS/MFG/VRC >32 mg/L, AMB/ISA 0.5 mg/L, 5-FC 0.06 mg/L (GDS)	NA	NA	NA	[26]
44	2008-2020 (Athens)	0.2 F	Congenital heart disease, renal failure, surgical procedure, detailed RF NA (NA)	*Saccharomyces cerevisiae* (MALDI-TOF MS, Bruker)	FLC 16 mg/L, ITC 4 mg/L, PSC 2 mg/L, CAS/ISA 0.125 mg/L, VRC 0.06 mg/L, AMB/MFG/5-FC 0.016 mg/L, AFG 0.008 mg/L (GDS)	NA	NA	NA	[26]
45	2008-2020 (Athens)	1.2 F	Pneumococcal meningitis, detailed RF NA (NA)	*Saccharomyces cerevisiae* (MALDI-TOF MS, Bruker)	FLC >256 mg/L, ITC >32 mg/L, PSC 8 mg/L, VRC 0.5 mg/L, CAS 0.25 mg/L, AMB 0.125 mg/L, AFG 0.06 mg/L, MFG/ISA 0.03 mg/L, 5-FC 0.016 mg/L (GDS)	NA	NA	NA	[26]
46	2008-2020 (Athens)	7 F	ALL, detailed RF NA (NA)	*Saccharomyces cerevisiae* (MALDI-TOF MS, Bruker)	FLC 4 mg/L, ITC 2 mg/L, PSC/5-FC 1 mg/L, MFG/CAS 0.25 mg/L, VRC 0.125 mg/L, AMB/ISA 0.03 mg/L, AFG 0.016 mg/L (GDS)	NA	NA	NA	[26]
47	2008-2020 (Athens)	0.8 F	Myeloblastoma, detailed RF NA (NA)	*Saccharomyces cerevisiae* (MALDI-TOF MS, Bruker)	FLC 16 mg/L, ITC 4 mg/L, PSC 2 mg/L, VRC/CAS 0.25 mg/L, ISA 0.125 mg/L, AMB/5-FC 0.06 mg/L, AFG/MFG 0.03 mg/L (GDS)	NA	NA	NA	[26
48	2008-2020 (Athens)	1 F	Detailed RF NA (NA)	*Saccharomyces cerevisiae* (MALDI-TOF MS, Bruker)	FLC/ITC 4 mg/L, PSC 2 mg/L, AMB/CAS 0.25 mg/L, MFG 0.06 mg/L, VRC/ISA 0.03 mg/L, AFG 0.016 mg/L, 5-FC 0.008 mg/L (GDS)	NA	NA	NA	[26]
49	2008-2020 (Athens)	0.6 F	Jejunal atresia, surgical procedure, detailed RF NA (NA)	*Saccharomyces cerevisiae* (MALDI-TOF MS, Bruker)	FLC 8 mg/L, ITC 4 mg/L, PSC 2 mg/L, AMB 0.5 mg/L, VRC/CAS 0.25 mg/L, ISA 0.06 mg/L, 5-FC/AFG/MFG 0.016 mg/L (GDS)	NA	NA	NA	[26]
50	2008-2020 (Athens)	2.5 F	Yolk sac malignancy, surgical procedure, detailed RF NA (NA)	*Trichosporon asahii* (MALDI-TOF MS, Bruker)	AFG/CAS/MFG/5-FC/AMB >32 mg/L, ITC 2 mg/L, PSC 0.5 mg/L, ISA 0.125 mg/L, VRC 0.06 mg/L (GDS)	NA	NA	NA	[26]
51	2008-2020 (Athens)	14 M	ALL, HSCT recipient, detailed RF NA (NA)	*Trichosporon asahii* (MALDI-TOF MS, Bruker)	AFG/CAS/MFG/5-FC/AMB >32 mg/L, FLC 16 mg/L, ITC 2 mg/L, PSC/ISA 0.5 mg/L, VRC 0.125 mg/L (GDS)	NA	NA	NA	[26]
52	2008-2020	10				NA	NA	NA	[26]

	(Athens)	F	Blackfan-Diamond anaemia, renal failure, detailed RF NA (NA)	*Trichosporon asahii* (MALDI-TOF MS, Bruker)	AFG/CAS/MFG/5-FC >32 mg/L, FLC/AMB 8 mg/L, ITC 4 mg/L, PSC 0.5 mg/L, ISA 0.125 mg/L (GDS)				
53	2018-2021 (Patras)	58 M	AML, CTX-induced myelotoxicity, CVC, detailed RF NA (neutropenic)	*Rhodotorula mucilaginosa* (MALDI-TOF MS, Bruker)	FLC >256 mg/L, AFG/CAS/MFG >32 mg/L, AMB/VRC/ITC 1 mg/L, PSC 0.25 mg/L, 5-FC 0.06 mg/L (GDS)	Yes (PSC, NA)	LAMB (NA)	Survival	[25]
54	2018-2021 (Patras)	19 M	T-cell ALL, CTX-induced myelotoxicity, CVC, detailed RF NA (neutropenic)	*Rhodotorula mucilaginosa* (MALDI-TOF MS, Bruker)	FLC >256 mg/L, AFG/CAS/MFG >32 mg/L, AMB/VRC 2 mg/L, ITC 1 mg/L, PSC 0.5 mg/L, ISA 0.25 mg/L, 5-FC 0.03 mg/L (GDS)	Yes (PSC, NA)	LAMB (NA)	Survival	[25]
55	2018-2021 (Patras)	48 M	Fabry disease, gastrostomy, detailed RF NA (NA)	*Saccharomyces cerevisiae* (MALDI-TOF MS, Bruker)	FLC 8 mg/L, POS 4 mg/L, ITC 2 mg/L, AMB/ISA 0.25 mg/L, 5-FC/VRC/AFG/CAS 0.125 mg/L, MFG 0.06 mg/L (GDS)	No	LAMB (NA)	Survival	[25]
56	2018-2021 (Patras)	71 F	Chronic heart failure, detailed RF NA (NA)	*Saccharomyces cerevisiae* (MALDI-TOF MS, Bruker)	ITC >32 mg/L, FLC 32 mg/L, POS 8 mg/L, AMB/VRC/ISA 0.5 mg/L, AFG 0.125 mg/L, MFG/CAS 0.06 mg/L, 5-FC 0.03 mg/L (GDS)	No	No	Death*	[25]
57	2018-2021 (Patras)	68 M	Reflux nephropathy and bladder malignancy, CVC, detailed RF NA (NA)	*Trichosporon asahii* (MALDI-TOF MS, Bruker)	AFG/CAS/MFG >32 mg/L, FLC 2 mg/L, ITC 1 mg/L, PSC 0.5 mg/L, AMB/5-FC 0.25 mg/L, ISA 0.125 mg/L, VRC 0.03 mg/L (GDS)	No	VRC (NA)	Survival	[25]
58	2018-2021 (Patras)	67 M	Traumatic brain injuries, CVC, detailed RF NA (NA)	*Trichosporon asahii* (MALDI-TOF MS, Bruker)	AFG/CAS/MFG >32 mg/L, 5-FC 4 mg/L, FLC 2 mg/L, AMB/ITC/PSC 0.5 mg/L, ISA 0.06 mg/L, VRC 0.03 mg/L (GDS)	Yes (AFG, dose NA x 17 days)	VRC (NA)	Survival	[25]
59	2018-2021 (Patras)	28 M	Skull base surgery, CVC, detailed RF NA (NA)	*Trichosporon asahii* (MALDI-TOF MS, Bruker)	AFG/CAS/MFG >32 mg/L, 5-FC 16 mg/L, FLC 2 mg/L, ITC 1 mg/L, AMB/PSC 0.25 mg/L, VRC/ISA 0.06 mg/L (GDS)	No	VRC (NA)	Survival	[25]
60	2018-2021 (Patras)	73 M	MDS/mixed-type ALL, CTX-induced myelotoxicity, CVC, detailed RF NA (neutropenic)	*Trichosporon asahii* (MALDI-TOF MS, Bruker)	AFG/CAS/MFG >32 mg/L, 5-FC 8 mg/L, FLC 2 mg/L, ITC/PSC 0.5 mg/L, ISA 0.125 mg/L, AMB/VRC 0.06 mg/L (GDS)	Yes (PSC, NA)	LAMB + VRC (NA)	Death	[25]
61	2018-2021 (Patras)	62 M	Neurosurgical procedure, CVC, detailed RF NA (NA)	*Trichosporon asahii* (MALDI-TOF MS, Bruker)	AFG/CAS/MFG >32 mg/L, FLC 4 mg/L, 5-FC 1 mg/L, ITC/PSC 0.5 mg/L, AMB 0.25 mg/L, VRC 0.06 mg/L, ISA 0.03 mg/L (GDS)	No	VRC (NA)	Survival	[25]
62	2018-2021 (Patras)	72 M	NHL, CTX-induced myelotoxicity, CVC, detailed RF NA (NA)	*Trichosporon asahii* (MALDI-TOF MS, Bruker)	AFG/CAS/MFG >32 mg/L, 5-FC 16 mg/L, FLC/ITC 1 mg/L, AMB/PSC 0.25 mg/L, ISA 0.125 mg/L, VRC 0.03 mg/L (GDS)	No	VRC (NA)	Survival	[25]
63	2018-2021 (Patras)	65 M	AML, CTX-induced myelotoxicity, CVC, detailed RF NA (neutropenic)	*Trichosporon asahii* (MALDI-TOF MS, Bruker)	AFG/CAS/MFG >32 mg/L, FLC 2 mg/L, ITC/PSC 1 mg/L, AMB/5-FC 0.5 mg/L, ISA 0.125 mg/L, VRC 0.06 mg/L (GDS)	Yes (PSC, NA)	VRC (NA)	Survival	[25]
64	2022 (Athens)	0.6 F	VLBW, RDS, Gram-negative BSI, CVC, ABT (non-neutropenic)	*Moesziomyces aphidis* (ITS sequencing)	AFG/MFG/CAS >8 mg/L, FLC 8 mg/L, VRC/PSC 0.03 mg/L, AMB ≤0.125 mg/L, ISA 0.016 mg/L, ITC ≤0.016 mg/L (EUCAST BMD)	No	LAMB (7 mg/kg/d x 31 days)	Survival	[12]

*Patient died before notification of positive blood culture.

Abbreviations. ABT, antibiotic treatment; AFG, anidulafungin; ALL, acute lymphoblastic leukaemia; ALS, amyotrophic lateral sclerosis; AMB, amphotericin B; AML, acute myeloid leukaemia; bid, twice daily; BMD, broth microdilution; BSI, bloodstream infection; CAS, caspofungin; CLSI, clinical and laboratory standards institute; COPD, chronic obstructive pulmonary disease; COVID-19, coronavirus disease 2019; CTX, chemotherapy; CVC, central venous catheter; ELBW, extremely low birth weight; EUCAST, European committee on antimicrobial susceptibility testing; F, female; FLC, fluconazole; GDS, gradient diffusion strip; HL, Hodgkin's lymphoma; HSCT, haematopoietic stem cell transplant; ISA, isavuconazole; ITC, itraconazole; ITS, internal transcribed spacer; LAMB, liposomal amphotericin B; M, male; MALDI-TOF MS, matrix-assisted laser desorption/ionization-time of flight mass spectrometry; MDS, myelodysplastic syndrome; MFG, micafungin; MRSA, methicillin-resistant *Staphylococcus aureus*; NA, not available; NHL, non-Hodgkin's lymphoma; No, number; po, orally; PN, parenteral nutrition; PSC, posaconazole; PVC, peripheral venous catheter; RDS, respiratory distress syndrome; RF, risk factors; SBP, *S. cerevisiae var. boulardii*-based probiotics; VLBW, very low birth weight; VRC, voriconazole; 5-FC, 5-flucytosine.

Supplementary Table S2. Reported cases of fungaemia caused by rare yeasts in "Attikon" University General Hospital (2010-2024), presented chronologically.

No	Diagnosis date	Age (years) Sex	Underlying conditions and risk factors (immune status)	Pathogen (identification method)	Antifungal susceptibility based on EUCAST BMD	Breakthrough infection (prior antifungal, duration)	Antifungal therapy	Outcome
1	June 2010	65 M	NHL, CVC, ABT (immunosuppressed)	*Rhodotorula mucilaginosa* (Vitek 2 YST)	NA	NA	NA	Survival
2	June 2011	83 F	Gastric malignancy, Gram-negative BSI, PN, CVC, ABT (immunosuppressed)	*Rhodotorula mucilaginosa* (MALDI-TOF MS, Bruker)	FLC >64 mg/L, AFG/CAS/MFG >8 mg/L, PSC/VRC 1 mg/L, ITC/ISA 0.5 mg/L, AMB 0.25 mg/L	No	FLC (200 mg/d x 9 days) → LAMB (5 mg/kg/d x 7 days)	Death
3	September 2011	84 F	Renal malignancy, haemodialysis, CVC, ABT (immunosuppressed)	*Rhodotorula mucilaginosa* (MALDI-TOF MS, Bruker)	FLC >64 mg/L, AFG/CAS/MFG >8 mg/L, VRC 4 mg/L, ISA/PSC 2 mg/L, ITC 1 mg/L, AMB 0.25 mg/L	No	LAMB (5 mg/kg/d x 30 days)	Survival
4	October 2011	66 F	Diabetes mellitus, PN, SBP, corticosteroid therapy, CVC, ABT (immunosuppressed)	*Saccharomyces cerevisiae* (MALDI-TOF MS, Bruker)	FLC 8 mg/L, ISA 1 mg/L, CAS/ITC 0.5 mg/L, PSC/AMB 0.25 mg/L, VRC 0.125 mg/L, AFG/MFG 0.06 mg/L	No	AFG (100 mg/d x 6 days)	Death
5	June 2012	25 M	Oesophageal malignancy, PN, CVC, ABT (immunosuppressed)	*Rhodotorula mucilaginosa* (MALDI-TOF MS, Bruker)	FLC >64 mg/L, AFG/CAS/MFG >8 mg/L, PSC/VRC 1 mg/L, ITC/ISA 0.5 mg/L, AMB 0.25 mg/L	Yes (FLC 400 mg/d x 25 days)	FLC (400 mg/d x NA days)	Death
6	March 2013	39 F	Burkitt's lymphoma, CVC, ABT (immunosuppressed)	*Rhodotorula mucilaginosa* (MALDI-TOF MS, Bruker)	FLC 32 mg/L, CAS >8 mg/L AFG/MFG 4 mg/L, PSC/VRC 2 mg/L, AMB/ITC 0.5 mg/L, ISA 0.25 mg/L	Yes (FLC 400 mg/d x 11 days)	FLC (400 mg/d x 5 days) → LAMB (5 mg/kg/d x 19 days)	Survival
7	September 2013	49 F	Breast malignancy, CVC, ABT (immunosuppressed)	*Rhodotorula mucilaginosa* (MALDI-TOF MS, Bruker)	FLC >64 mg/L, AFG/CAS/MFG >8 mg/L, PSC/VRC 1 mg/L, ITC/ISA 0.5 mg/L, AMB 0.25 mg/L	NA	NA	Death
8	May 2014	70 F	NHL, detailed RF NA (immunosuppressed)	*Saccharomyces cerevisiae* (MALDI-TOF MS, Bruker)	FLC 16 mg/L, ITC 8 mg/L, CAS/PSC/ISA 0.5 mg/L, AMB 0.25 mg/L, AFG/MFG/VRC 0.125 mg/L	NA	NA	Death
9	May 2014	84 M	Brain malignancy, surgical procedure, prolonged ICU stay, PN, CVC, ABT (immunosuppressed)	*Trichosporon asahii* (MALDI-TOF MS, Bruker)	MFG/CAS >8 mg/L, FLC 8 mg/L, AFG 4 mg/L, AMB 1 mg/L, PSC/ITC 0.5 mg/L, VRC 0.125 mg/L, ISA 0.06 mg/L	No	LAMB (5 mg/kg/d x 3 days) → LAMB (5 mg/kg/d) + VRC (4 mg/kg bid) x 4 days	Death
10	May 2015	17 M	Severe ankle injury, surgical procedure, PN, SBP, CVC, ABT (non-neutropenic)	*Saccharomyces cerevisiae* (Vitek 2 YST)	NA	No	AFG (100 mg/d x 31 days)	Survival
11	July 2015	69 M	Small bowel perforation, liver cirrhosis, abdominal surgery, PN, CVC, ABT (non-neutropenic)	*Rhodotorula mucilaginosa* (Vitek 2 YST)	NA	No	AFG (100 mg/d x 2 days) → LAMB (5 mg/kg/d x 20 days)	Survival
12	January 2017	69 M	Pulmonary malignancy, PN, SBP, CVC, ABT (immunosuppressed)	*Saccharomyces cerevisiae* (MALDI-TOF MS, Bruker)	ITC >8 mg/L, FLC 8 mg/L, CAS/PSC/ISA 1 mg/L, AMB 0.25 mg/L, AFG/MFG/VRC 0.125 mg/L	No	FLC (400 mg/d x 6 days) → CAS (50 mg/d x 4 days)	Death
13	January 2017	81 M	Parkinson's disease, PN, SBP, CVC, ABT (non-neutropenic)	*Saccharomyces cerevisiae* (Vitek 2 YST)	NA	No	No	Death*
14	October 2017	77 M	CLL, CVC, ABT (immunosuppressed)	*Rhodotorula mucilaginosa* (MALDI-TOF MS, Bruker)	FLC >64 mg/L, AFG/CAS/MFG >8 mg/L, VRC 2 mg/L, PSC 1 mg/L, ITC 0.5 mg/L, AMB/ISA 0.25 mg/L	NA	NA	Survival
15	October 2017	67 M	AML, detailed RF NA (neutropenic)	*Trichosporon asahii* (MALDI-TOF MS, Bruker)	AFG/MFG/CAS >8 mg/L, FLC 16 mg/L, AMB 1 mg/L, PSC/ITC 0.5 mg/L, VRC 0.25 mg/L, ISA 0.125 mg/L	NA	NA	Death
16	February 2018	67 M	Endocarditis, detailed RF NA (non-neutropenic)	*Rhodotorula mucilaginosa* (MALDI-TOF MS, Bruker)	FLC >64 mg/L, AFG/CAS/MFG >8 mg/L, VRC 2 mg/L, ITC/PSC 0.5 mg/L, AMB/ISA 0.25 mg/L	No	No	Death*
17	May	53		*Saccharomyces cerevisiae*		NA	NA	Death

	2018	F	Respiratory failure, detailed RF NA (non-neutropenic)	(MALDI-TOF MS, Bruker)	FLC 16 mg/L, ITC 8 mg/L, PSC/ISA 1 mg/L, CAS 0.5 mg/L, AMB 0.25 mg/L, AFG/MFG/VRC 0.125 mg/L			
18	May 2018	78 F	Autoimmune hepatitis, diabetes mellitus, abdominal surgery, PN, CVC, ABT (immunosuppressed)	*Saccharomyces cerevisiae* (MALDI-TOF MS, Bruker)	FLC 16 mg/L, ISA 1 mg/L, CAS/ITC/PSC 0.5 mg/L, AMB 0.25 mg/L, AFG/MFG/VRC 0.125 mg/L	No	CAS (50 mg/d x NA days)	Survival
19	July 2018	77 M	Diabetes mellitus, abdominal surgery, PN, CVC, ABT (non-neutropenic)	*Saccharomyces cerevisiae* (MALDI-TOF MS, Bruker)	ITC >8 mg/L, FLC 8 mg/L, ISA/PSC 1 mg/L, CAS 0.5 mg/L, AMB 0.25 mg/L, MFG/VRC 0.125 mg/L, AFG 0.06 mg/L	Yes (AFG 100 mg/d x 23 days)	AFG (100 mg/d x 25 days)	Survival
20	December 2018	74 F	Lower respiratory infection, detailed RF NA (non-neutropenic)	*Trichosporon asahii* (MALDI-TOF MS, Bruker)	FLC 16 mg/L, AFG/MFG/CAS 4 mg/L, AMB 1 mg/L, PSC 0.25 mg/L, ITC 0.125 mg/L, VRC/ISA 0.06 mg/L	NA	NA	Death
21	July 2020	64 M	Breast malignancy with liver metastases, CVC, ABT (immunosuppressed)	*Rhodotorula mucilaginosa* (MALDI-TOF MS, Bruker)	FLC >64 mg/L, AFG/CAS/MFG >8 mg/L, VRC 2 mg/L, ITC/PSC 1 mg/L, AMB/ISA 0.25 mg/L	NA	NA	Survival
22	January 2022	59 M	Diabetes mellitus, diabetic foot, COVID-19, Gram-negative BSI, CVC, ABT (non-neutropenic)	*Trichosporon asahii* (MALDI-TOF MS, Bruker)	AFG/MFG/CAS >8 mg/L, FLC 8 mg/L, AMB 1 mg/L, PSC/ITC 0.5 mg/L, ISA 0.125 mg/L, VRC 0.06 mg/L	No	LAMB (5 mg/kg/d x 10 days) → VRC (4 mg/kg bid x 21 days)	Survival
23	March 2022	88 M	Extensive facial and chest burns, prolonged ICU stay, diabetes mellitus, PN, CVC, ABT (non-neutropenic)	*Trichosporon asahii* (MALDI-TOF MS, Bruker)	MFG/CAS >8 mg/L, FLC 8 mg/L, AFG 4 mg/L, AMB 1 mg/L, PSC 0.5 mg/L, ITC 0.25 mg/L, ISA 0.125 mg/L, VRC 0.06 mg/L	Yes (AFG 100 mg/d x 9 days)	AFG (100 mg/d x 5 days)	Death
24	November 2022	59 M	AML, CTX, diabetes mellitus, PN, CVC, ABT (neutropenic)	*Trichosporon asahii* (MALDI-TOF MS, Bruker)	MFG/CAS >8 mg/L, FLC 16 mg/L, AFG 4 mg/L, AMB 1 mg/L, PSC/ITC/ISA 0.5 mg/L, VRC 0.25 mg/L	No	LAMB (5 mg/kg/d) + VRC (4 mg/kg bid) x 17 days	Death
25	January 2024	62 F	AML, HSCT recipient, CTX, CVC, ABT (neutropenic)	*Apiotrichum loubieri* (MALDI-TOF MS, Bruker)	MFG/CAS >8 mg/L, AFG 4 mg/L, FLC 1 mg/L, AMB 0.25 mg/L, VRC 0.125 mg/L, PSC/ISA 0.03 mg/L, ITC 0.016 mg/L	Yes (ISA 200 mg/d x 22 days)	LAMB (5 mg/kg/d x 4 days)	Death
26	January 2024	75 M	Refractory AML, CTX, diabetes mellitus, CVC, ABT (neutropenic)	*Rhodotorula mucilaginosa* (MALDI-TOF MS, Bruker)	FLC >64 mg/L, AFG/CAS/MFG >8 mg/L, VRC 2 mg/L, ITC/PSC 1 mg/L, AMB/ISA 0.25 mg/L	No	LAMB (5 mg/kg/d x 5 days)	Death
27	March 2024	36 M	AML, HSCT recipient, CTX, CVC, ABT (neutropenic)	*Rhodotorula mucilaginosa* (MALDI-TOF MS, Bruker)	FLC >64 mg/L, AFG/CAS/MFG >8 mg/L, VRC 2 mg/L, ITC/PSC 1 mg/L, AMB/ISA 0.5 mg/L	Yes (ISA 200 mg/d x 14 days)	LAMB (5 mg/kg/d x 27 days)	Survival
28	June 2024	74 M	*Clostridium difficile*-associated enterocolitis, PN, SBP, CVC, ABT (non-neutropenic)	*Saccharomyces cerevisiae* (MALDI-TOF MS, Bruker)	FLC 16 mg/L, ITC/PSC 1 mg/L, AMB/CAS 0.5 mg/L, VRC/ISA/AFG 0.125 mg/L, MFG 0.06 mg/L	No	No	Death*
29	August 2024	77 F	Polytrauma, prolonged ICU stay, PN, CVC, ABT (non-neutropenic)	*Magnusiomyces capitatus*# (ITS sequencing)	FLC 8 mg/L, CAS 4 mg/L, AFG/MFG 2 mg/L, AMB/ITC 0.5 mg/L, VRC/PSC/ISA 0.25 mg/L	Yes (AFG 100 mg/d x 14 days, escalated to 200 mg/d x 5 days)	No	Death*

*Patient died before notification of positive blood culture.

#Mixed fungaemia with *Candida parapsilosis* (persistent candidaemia).

Abbreviations. ABT, antibiotic treatment; AFG, anidulafungin; AMB, amphotericin B; AML, acute myeloid leukaemia; bid, twice daily; BMD, broth microdilution; BSI, bloodstream infection; CAS, caspofungin; CLL, chronic lymphocytic leukaemia; COVID-19, coronavirus disease 2019; CTX, chemotherapy; CVC, central venous catheter; EUCAST, European committee on antimicrobial susceptibility testing; F, female; FLC, fluconazole; HSCT, haematopoietic stem cell transplant; ICU, intensive care unit; ISA, isavuconazole; ITC, itraconazole; ITS, internal transcribed spacer; LAMB, liposomal amphotericin B; M, male; MALDI-TOF MS, matrix-assisted laser desorption/ionization-time of flight mass spectrometry; MFG, micafungin; NA, not available; NHL, non-Hodgkin's lymphoma; No, number; PN, parenteral nutrition; PSC, posaconazole; RF, risk factors; SBP, *S. cerevisiae var. boulardii*-based probiotics; VRC, voriconazole.

REFERENCES

21. Papadogeorgakis, H.; Frangoulis, E.; Papaefstathiou, C.; Katsambas, A. *Rhodotorula Rubra* Fungaemia in an Immunosuppressed Patient. *J. Eur. Acad. Dermatology Venereol.* **1999**, *12*, 169–170, doi:10.1111/J.1468-3083.1999.TB01010.X.

20. Petrocheilou-Paschou, V.; Prifti, H.; Kostis, E.; Papadimitriou, C.; Dimopoulos, M.A.; Stamatelopoulos, S. Rhodotorula Septicemia: Case Report and Minireview. *Clin. Microbiol. Infect.* **2001**, *7*, 100–102, doi:10.1046/j.1469-0691.2001.00203.x.

19. Samonis, G.; Anatoliotaki, M.; Apostolakou, H.; Maraki, S.; Mavroudis, D.; Georgoulias, V. Transient Fungemia Due to *Rhodotorula Rubra* in a Cancer Patient: Case Report and Review of the Literature. *Infection* **2001**, *29*, 173–176, doi:10.1007/S15010-001-1066-1/METRICS.

18. Panagopoulou, P.; Evdoridou, J.; Bibashi, E.; Filioti, J.; Sofianou, D.; Kremenopoulos, G.; Roilides, E. *Trichosporon Asahii*: An Unusual Cause of Invasive Infection in Neonates. *Pediatr. Infect. Dis. J.* **2002**, *21*, 169–170, doi:10.1097/00006454-200202000-00018,.

17. Christakis, G.; Perlorentzou, S.; Aslanidou, M.; Megalakaki, A.; Velegraki, A. Fatal *Blastoschizomyces Capitatus* Sepsis in a Neutropenic Patient with Acute Myeloid Leukemia: First Documented Case from Greece. *Mycoses* **2005**, *48*, 216–220, doi:10.1111/J.1439-0507.2005.01098.X.

16. Antachopoulos, C.; Papakonstantinou, E.; Dotis, J.; Bibashi, E.; Tamiolaki, M.; Koliouskas, D.; Roilides, E. Fungemia Due to *Trichosporon Asahii* in a Neutropenic Child Refractory to Amphotericin B: Clearance with Voriconozole. *J. Pediatr. Hematol. Oncol.* **2005**, *27*, 283–285, doi:10.1097/01.MPH.0000164865.70522.D7,.

22. Kofteridis, D.; Mantadakis, E.; Christidou, A.; Samonis, G. *Rhodotorula Glutinis* Fungemia Successfully Treated with Fluconazole: Report of Two Cases. *Int. J. Infect. Dis.* **2007**, *11*, 179–180, doi:10.1016/j.ijid.2006.02.004.

15. Spiliopoulou, A.; Anastassiou, E.D.; Christofidou, M. *Rhodotorula* Fungemia of an Intensive Care Unit Patient and Review of Published Cases. *Mycopathologia* **2012**, *174*, 301–309, doi:10.1007/S11046-012-9552-9/METRICS.

14. Vrioni, G.; Chronopoulou, G.; Vossou, C.; Theodoridou, K. *Saprochaete Capitata* (Former Name of *Geotrichum Capitatum*) Fungemia in a Patient with Chronic Neurological and Hematological Disease. *Acta Microbiol. Hell.* **2017**, *62*, 109–114.

24. Siopi, M.; Tarpatzi, A.; Kalogeropoulou, E.; Damianidou, S.; Vasilakopoulou, A.; Vourli, S.; Pournaras, S.; Meletiadis, J. Epidemiological Trends of Fungemia in Greece with a Focus on Candidemia during the Recent Financial Crisis: A 10-Year Survey in a Tertiary Care Academic Hospital and Review of Literature. *Antimicrob. Agents Chemother.* **2020**, *64*, e01516-19, doi:10.1128/AAC.01516-19.

13. Gkentzi, D.; Marangos, M.; Karatza, A.; Spiliopoulou, A.; Varvarigou, A.; Dimitriou, G. *Saccharomyces cerevisiae* fungaemia in an immunocompetent toddler. *J. Paediatr. Child Health* **2020**, *56*, 182–182, doi:10.1111/JPC.14740.

23. Ventoulis, I.; Sarmourli, T.; Amoiridou, P.; Mantzana, P.; Exindari, M.; Gioula, G.; Vyzantiadis, T.A. Bloodstream Infection by *Saccharomyces Cerevisiae* in Two COVID-19 Patients after Receiving Supplementation of Saccharomyces in the ICU. *J. Fungi* **2020**, *6*, 98, doi:10.3390/JOF6030098.

26. Noni, M.; Stathi, A.; Velegraki, A.; Mika, M.; Kalampaliki, A.; Zachariadou, L.; Michos, A. Rare Invasive Yeast Infections in Greek Neonates and Children, a Retrospective 12-Year Study. *J. Fungi* **2020**, *6*, 1–13, doi:10.3390/jof6040194.

25. Spiliopoulou, A.; Lekkou, A.; Vrioni, G.; Leonidou, L.; Cogliati, M.; Christofidou, M.; Marangos, M.; Kolonitsiou, F.; Paliogianni, F. Fungemia Due to Rare Non-*Candida* Yeasts between 2018 and 2021 in a Greek Tertiary Care University Hospital. *J. Med. Mycol.* **2023**, *33*, 101386, doi:10.1016/J.MYCMED.2023.101386.

12. Mpakosi, A.; Siopi, M.; Demetriou, M.; Falaina, V.; Theodoraki, M.; Meletiadis, J. Fungemia Due to *Moesziomyces Aphidis* (*Pseudozyma Aphidis*) in a Premature Neonate. Challenges in Species Identification and Antifungal Susceptibility Testing of Rare Yeasts. *J. Med. Mycol.* **2022**, *32*, 101258, doi:10.1016/J.MYCMED.2022.101258.
